# Supplementary material for: Association between Age-Friendliness of Communities and Frailty among Older Adults: A Multilevel Analysis
Source: Int J Environ Res Public Health. 2022 Jun 20;19(12):7528. doi: 10.3390/ijerph19127528 (PMC9224492; doi:10.3390/ijerph19127528)
Supplement: Supplementary file 1 [file ijerph-19-07528-s001.zip › ijerph-1753651-supplementary.pdf]

Table S1: Age-friendly Community Evaluation Scale. (Residential environment: The following questions are about your neighborhoods and the description of environment related problems, please according to your actual feeling, choose one of the following options: 1: completely disagree, 2: disagree, 3: generally, neither agree nor disagree (neutral), 4: agree, 5: completely agree).

**A Housing**					
1. No financial pressure on housing (e.g. mortgage, rent, etc.)	1	2	3	4	5
2. The water, electricity and coal of the house are well equipped and can be used normally	1	2	3	4	5
3. The housing can meet the needs of safety, convenience, comfort and hygiene (such as anti-skid floor, handrails, ramps or elevators for wheelchair access, etc.)	1	2	3	4	5
**B Transportation**					
1. There are safe and reliable public transportation, convenient for going out	1	2	3	4	5
2.There are many public transportations, the carriage is not crowded, and there are special seats for the elderly, the sick, the disabled and pregnant	1	2	3	4	5
3. Even in places inaccessible by public transport, there are convenient means of transportation such as free shuttle buses	1	2	3	4	5
4. There are enough seats for the elderly in buses and subways (other people will offer seats for the elderly when there are not enough seats for them)	1	2	3	4	5
5. The bus runs smoothly, and the driver makes sure that it is safe to leave after the stop	1	2	3	4	5
6. It is easy to get to the destination by public transport (e.g. the bus route and timetable at the station are clearly indicated, and the boarding and boarding are safe and barrier-free, etc.)	1	2	3	4	5
7. The bus station has suitable hardware facilities (such as seats, awnings, etc.), and the waiting environment is comfortable	1	2	3	4	5
**C Built Environment**					
1. Community public Spaces (such as roads, parks, etc.) are clean, tidy and well maintained	1	2	3	4	5
2. It's easy to walk or park from my house	1	2	3	4	5
3.The pavement surface is smooth, without obstacles, and it is safe to walk	1	2	3	4	5
4.The traffic lights near the community give pedestrians enough time to cross the road	1	2	3	4	5
5.On the road, motor vehicles (such as cars, lorries, etc.) can yield to pedestrians	1	2	3	4	5

6. On the road, non-motor vehicles (such as electric cars, bicycles, etc.) can yield to pedestrians	1	2	3	4	5
**D Social Participation**					
1. It is very convenient to get to places for leisure and cultural activities in the community	1	2	3	4	5
2. The community will choose leisure and cultural activities at times convenient to us	1	2	3	4	5
3. Easy access to information about community activities (e.g. participation modes, equipment using methods, transportation routes, etc.)	1	2	3	4	5
4. Various leisure and sports activities are carried out in the community (such as singing, calligraphy, chess, sports competition, popular science lectures, etc.)	1	2	3	4	5
5.There are many volunteer opportunities in the community	1	2	3	4	5
6.The government and the community will provide continuous help and support to those in need	1	2	3	4	5
**E Social Inclusion and Health Services**					
1. People in the community are respectful and polite to the elderly	1	2	3	4	5
2. Staff in municipal, district, sub-district offices and public health institutions are friendly and helpful	1	2	3	4	5
3.Community activities cater to the needs and preferences of people of all ages and are attractive	1	2	3	4	5
4. I feel respected in my social interactions	1	2	3	4	5
5. To better serve the elderly, relevant departments and organizations regularly solicit opinions from the elderly	1	2	3	4	5
6. The mass media (television, news, newspapers, radio programs, etc.) portray older people in a positive way	1	2	3	4	5
7. Older persons have easy and safe access to health facilities and public service facilities (e.g. centers for the elderly, activity rooms for the elderly, etc.)	1	2	3	4	5
8. Staff in health facilities and public services are helpful	1	2	3	4	5
9. Where I live, I have easy access to information on health education, nutrition courses and physical therapy	1	2	3	4	5
10. In public institutions (e.g. city, district, sub-district offices, etc.), Internet access is free or for a small fee	1	2	3	4	5

Table S2: Chinese frailty screening-10 (CFS-10). The following information is about your physical condition. Please check it and choose the actual option according to the description.

Items	Options	
(1) Do you have 5 or more chronic diseases? (hypertension, diabetes, stroke, cerebral infarction, cerebral hemorrhage), ischemic heart disease, chronic lung disease, tumor / cancer (except for mild skin cancer), congestive heart failure, angina, asthma, arthritis, kidney disease, cirrhosis, chronic liver disease, gastrointestinal diseases, Parkinson, musculoskeletal disorders;	1.Yes	2.No
(2) Have you often felt tired in the last month? (tired, weak, weak, exhausted);	1.Yes	2.No
(3) Has your food intake decreased in the last three months? (due to anorexia, dyspepsia, bad mouth or dysphagia);	1.Yes	2.No
(4) Has your life been affected by vision problems?	1.Yes	2.No
(5) Has your life been affected by hearing problems?	1.Yes	2.No
(6) Do you find it difficult to climb ten steps or one floor without a break or the help of any auxiliary tools?	1.Yes	2.No
(7) Have you walked for 10 minutes or 400 meters in the past week?	1.Yes	2.No
(8) Have you been distracted or hard to concentrate in the last month?	1.Yes	2.No
(9) Do you often get the wrong date or get lost in the last month?	1.Yes	2.No
(10) In the last month, have you done anything that you are not interested in?	1.Yes	2.No

Table S3: Description of mentioned Classification variables.

Variables	The Specific Classification
Age(years)	1='65~'; 2='70~'; 3='75~'; 4='80~';
Gender	1='Male'; 2='Female';
Marital status	2='In marriage'; other='Not in marriage'(including unmarried, divorced, and widowed);
Education level	1=' Below Primary School'; 2=' Primary School'; 3=' Junior high school'; 4-7=' High school and above';
Self-rated health	1-2='Poor or Worse'; 3='General'; 4='Better'; 5='Perfect';
Smoking	1='No'; 2='Used to'; 3='Smoking';
Drinking	1='No'; 2-5='Drinking';
Vegetable intake	1-2='Low'; 3-5='Adequate';
Fruit intake	1-2='Low'; 3-5='Adequate';
Physical activity	0='Inactive'; 1='Active';
Chronic disease	1='Without NCD'; 2='With one diseases'; 3='With Two or more';
Depression	0='No'; 1='Yes';
Cognitive Function	0='Normal'; 1='Cognitive decline';
Frail status	0='Robust'; 1='Frail';
Individual-level AFC Grade	1='First quartile'; 2='Second quartile'; 3='Third quartile'; 4='Fourth quartile';
Community-level AFC Grade	1='First quartile'; 2='Second quartile'; 3='Third quartile'; 4='Fourth quartile';
